# Supplementary material for: Src-transformed cells hijack mitosis to extrude from the epithelium
Source: Nat Commun. 2018 Nov 8;9:4695. doi: 10.1038/s41467-018-07163-4 (PMC6224566; doi:10.1038/s41467-018-07163-4)
Supplement: Supplementary file 1 — Supplementary Information [file 41467_2018_7163_MOESM1_ESM.pdf]

**Supplementary Information**

# Src-transformed cells hijack mitosis to extrude from the epithelium

**Anton et al.**

## Supplementary Figures

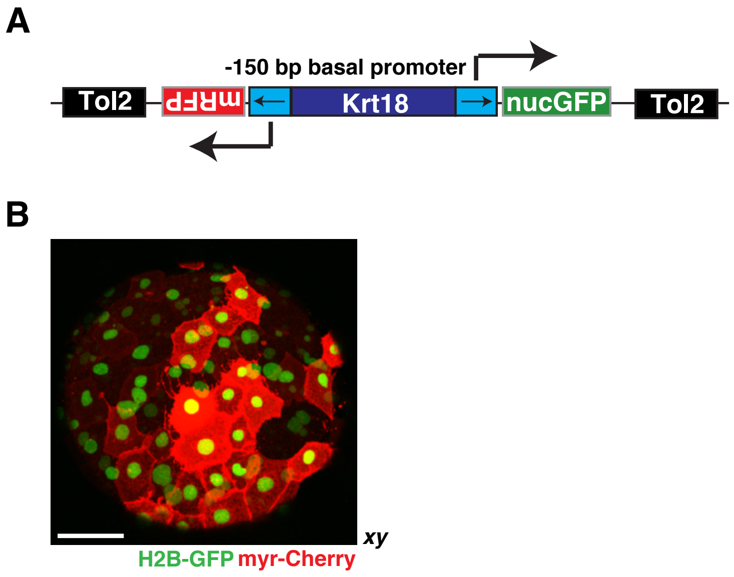


**Supplementary Figure 1. Establishment of bidirectional Krt18 promoter. (A)** The basal promoter region of the Krt18 promoter, -150 bp, is added to -5kb upstream of the initiation Methionine in the reverse-complement orientation. This bi-directional Krt18 promoter (dKrt18) can drive H2B-GFP and myr-Cherry only in the same EVL cells in a mosaic manner. **(B)** An example of expression of H2B-GFP and myr-Cherry at the tailbud stage. Wild-type embryos were injected with the dKrt18:H2B-GFP;myr-Cherry construct. Scale bar, 100 $\mu$m.

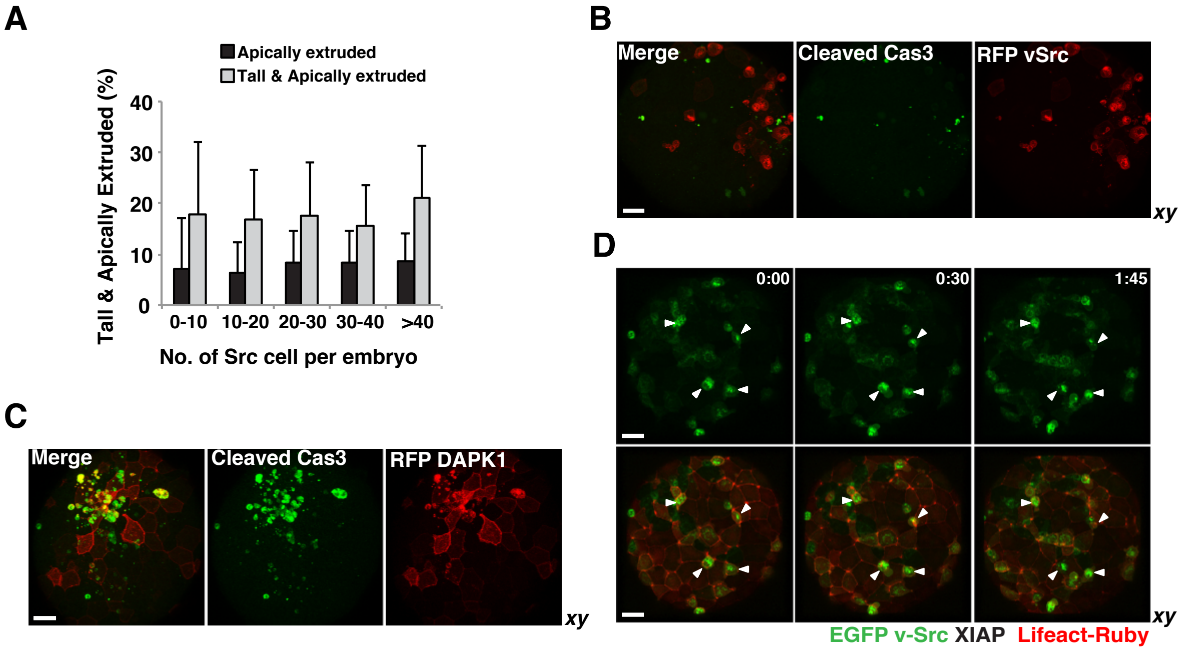


**Supplementary Figure 2. vSrc-driven extrusion is cell-death independent. (A)** Quantification of vSrc-driven cell extrusion and rounding rates based on cell density in fixed embryos. Embryos were injected with the dUAS:EGFP-vSrc. Data represent 34 independent experiments (number of embryos: $n_{Src}$ = 344). Error bars represent s.d. **(B)** Immunofluorescence images of Cleaved-Cas3 (green) in EVL cells expressing myr-Cherry-vSrc (red). Scale bar, 50 $\mu$m. **(C)** Immunofluorescence images of Cleaved-Cas3 (green) in EVL cells expressing myr-Cherry and DAPK1 (death-associated kinase 1; red). Scale bar, 50 $\mu$m. **(D)** The effect of inhibiting apoptosis in vSrc-mediated extrusion visualised in time-lapse imaging. Embryos obtained from the Krt18:Lifeact-Ruby) line expressing crossed with the Krt18:KalTA4-ERT2 line were injected with the dUAS:myr-Cherry-vSrc;XIAP construct (red). Movies were taken over 4 hours. Frames were extracted from a representative movie at indicated times from the tailbud stage. White arrowheads indicate cells that become extruded. Scale bars, 50 $\mu$m.

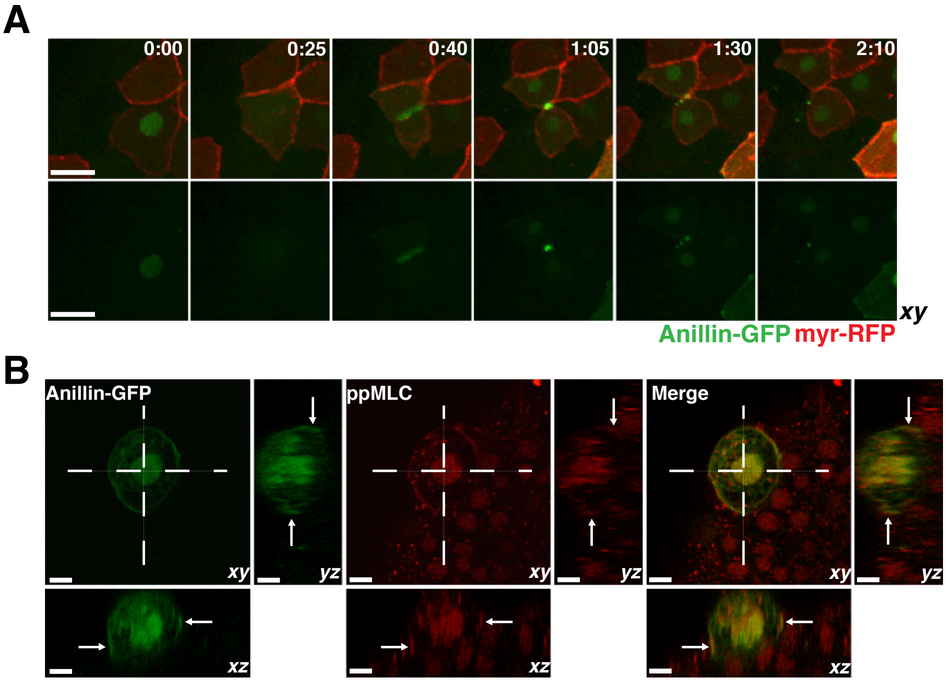


**Supplementary Figure 3. Analyses of the cytokinetic ring components in vSrc cell extrusion. (A)** Time-lapse imaging of Anillin-GFP in mitosis. Embryos were injected with the dUAS:myr-Cherry;Anillin-GFP construct. Movies were taken over 4 hours. Frames were extracted from a representative movie at indicated times from the tailbud stage. Scale bars, 25 $\mu$m. **(B)** Immunofluorescence images of phosphorylated myosin light chain (pMLC Thr18, Ser19; red) in the EVL cells expressing the Anillin-GFP (green) alongside vSrc. Scale bar, 10 $\mu$m. Arrows indicate lateral enrichment of Anillin-GFP.

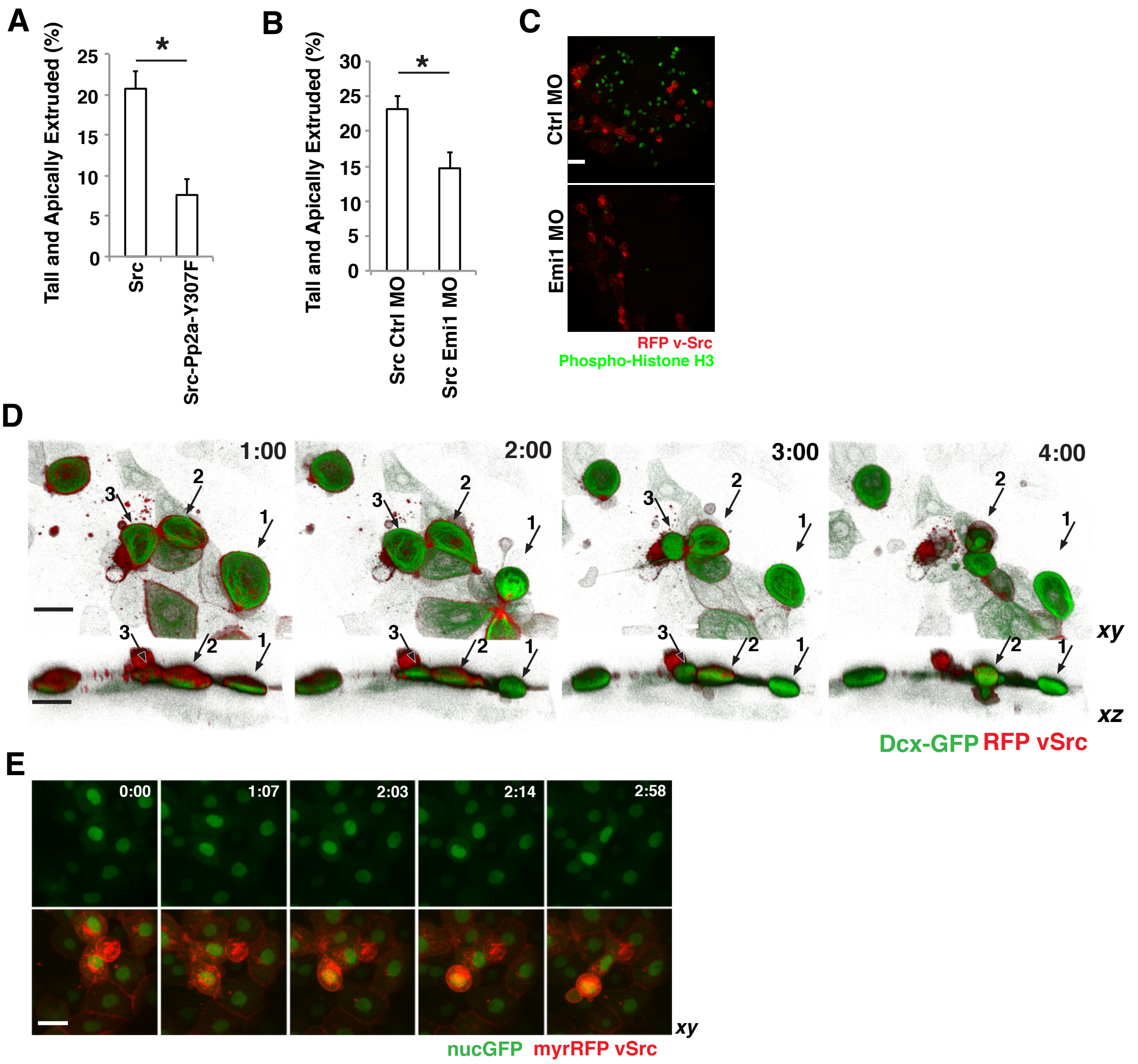


**Supplementary Figure 4. Analyses of mitotic markers in vSrc-driven extrusion.** **(A)** The effect of CA-Pp2a on vSrc-driven extrusion. Embryos were injected with the following constructs: dUAS:EGFP-vSrc and dUAS:EGFP-vSrc;Pp2a-Y307F (CA-Pp2a). Data are mean ± s.d. of 3 independent experiments (total number of embryos: $n_{Src} = 35$; $n_{Src,CA\text{-}pp2a} = 40$). $^*P < 0.05$ (Student's t-test). **(B)** The effect of *Emi1* morpholino on vSrc-driven extrusion. Embryos were injected with the UAS:EGFP-vSrc construct and the control or *Emi1* morpholinos (G2/M arrest[1]). Data are mean ± s.d. of 3 independent experiments (total number of embryos: $n_{Ctrl} = 15$; $n_{Emi1MO} = 15$). $^*P < 0.05$ (Student's t-test). **(C)** The effect of the *Emi1* MO on cell proliferation in the embryo. Embryos from (B) were fixed and stained for pH3 (green). A representative image was selected. Scale bar, 50 $\mu$m. **(D)** Time-lapse imaging of a microtubule marker Dcx-GFP in extrusion. Embryos were injected with the dUAS:myr-Cherry-vSrc;Dcx-GFP construct. Movies were taken over 4 hours. Frames were extracted from a representative movie at indicated from the tailbud stage ($t = 0$). Image is presented in the blend projection view of Imaris. Black arrows indicate cells that become extruded. Scale bars, 25 $\mu$m (*xy*) 25 $\mu$m (*xz*). **(E)** Time-lapse imaging of nucGFP in extrusion (3xNLS-Azami-GFP fusion). Embryos were injected with the dUAS:myr-Cherry-vSrc;nucGFP construct. Movies were taken over 4 hours. Frames were extracted from a representative movie at indicated from the tailbud stage ($t = 0$). Scale bars, 25 $\mu$m.

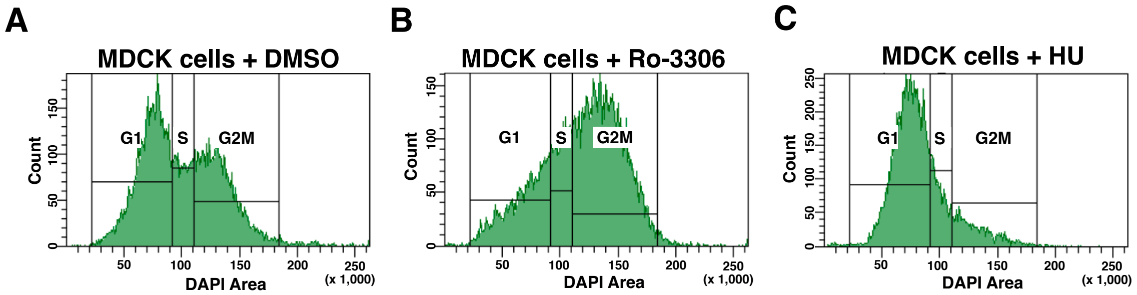


**Supplementary Figure 5. FACS analyses of the effect of proliferation inhibitors on DNA content in MDCK cells.** Prior to FACS analyses, MDCK populations were treated with DMSO (A), 10$\mu$M Ro-3306 (B), 2 mM hydroxyurea (C) and stained with DAPI.

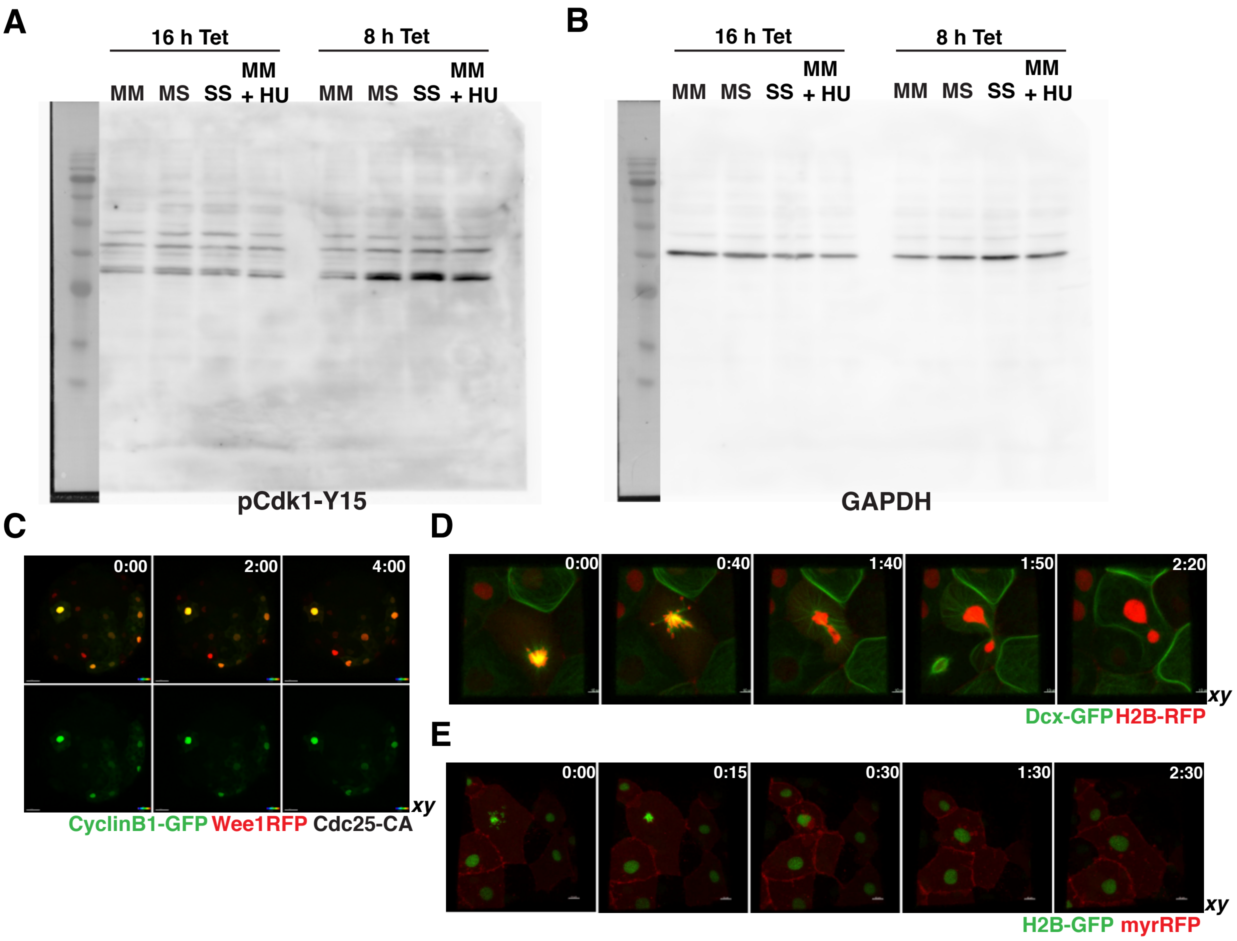


**Supplementary Figure 6. Analyses of cell cycle progression in MDCK cells and the EVL. (A, B)** The effect on phosphorylation of CDK1 after 16 and 8 hours from Src activation in MDCK cells. MM – MDCK cells alone, SS – Src cells alone, MS – cultures mixed 1:1, MM+HU – MDCK cells treated with 2mM hydroxyurea (HU). Original blots corresponding to Fig. 6C with pCDK staining (A) and GAPDH loading control (B). **(C)** Time-lapse imaging of CyclinB1-GFP localisation in cells arrested at the G2/M transition. Embryos were injected with a combination of the following constructs: dUAS:Cherry-Wee1;CA-Cdc25 and Krt18:CcnB1-GFP. Movies were taken over 4 hours. Frames were extracted from a representative movie at indicated times from the tailbud stage. Scale bars, 50 $\mu$m. **(D,E)** The effect of inducing monopolar spindles in the EVL in time-lapse imaging. Embryos were injected with the following constructs: dUAS:Dcx-GFP;H2B-RFP (D) and dUAS:myr-Cherry;H2B-GFP (E). At 50-70% epiboly embryos were treated for 3 hours with the Kif11 inhibitor, 0.875 mM STLC. Movies were taken over 4 hours in the presence of the inhibitor. Frames were extracted from a representative movie at indicated times from the tailbud stage. Scale bars, 10 $\mu$m.

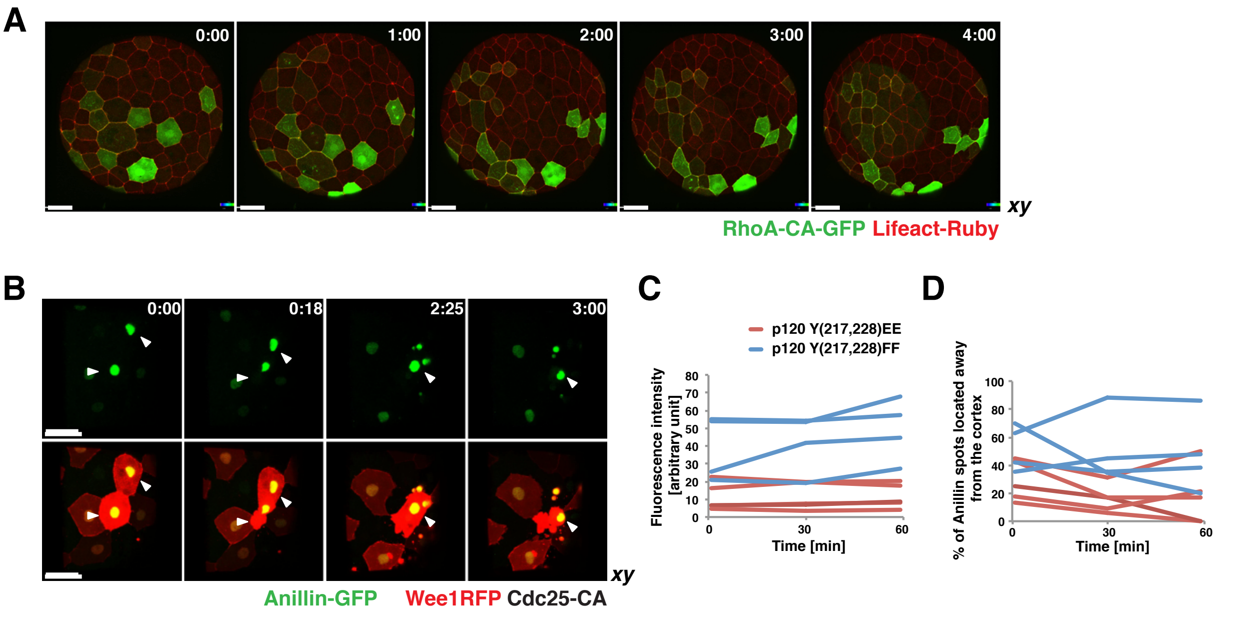


**Supplementary Figure 7. Determining conditions for misoriented cytokinetic ring assembly. (A)** The effect of RhoA activation in the EVL. Transgenic embryos obtained from a line expressing an RFP-actin marker (red) specifically in the EVL (Krt18:Lifeact-Ruby) line crossed with the Krt18:KalTA4-ERT2 line were injected with the dUAS:GFP-CAAX;CA-RhoA construct. Movies were taken over 4 hours. Frames were extracted from a representative movie at indicated times from the tailbud stage. Scale bars, 50 $\mu$m. **(B)** Time-lapse imaging of Anillin-GFP localization in cells arrested at the G2/M transition. Embryos were injected with a combination of the following constructs: dUAS:Cherry-Wee1;CA-Cdc25 and dUAS:myr-Cherry;Anillin-GFP. Movies were taken over 4 hours. Frames were extracted from a representative movie at indicated times from the tailbud stage. White arrowheads indicate cells that become extruded due to protein overexpression. Scale bars, 50 $\mu$m. **(C,D)** Quantification of the average fluorescence intensity (C) and position in regards to the cortex (D) of Anillin GFP spots over time in cells expressing either p120-mutEE or p120-mutFF. Embryos were injected with the following constructs: dUAS:Cherry-Wee1;CA-Cdc25 and either dUAS:p120-mutEE;AnillinGFP or dUAS:p120-mutFF;AnillinGFP. Data from 4 cells p120-mutFF (in blue) and 5 cells for p120-mutFF (in red) were acquired in 6 independent experiments and plotted.

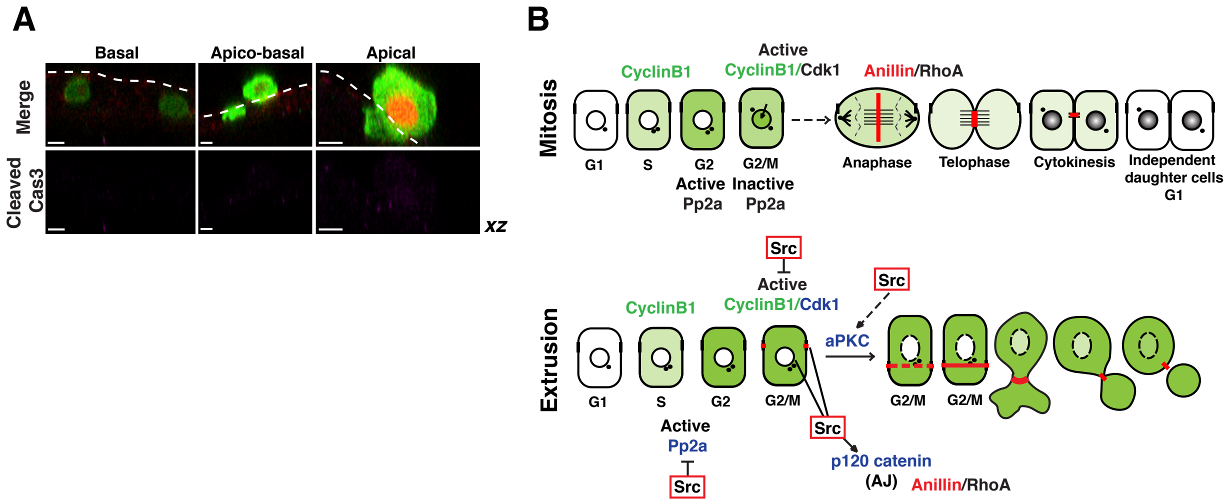


**Supplementary Figure 8. Key conditions for vSrc-mediated extrusion. (A)** Immunofluorescence images of Cleaved-Cas3 (magenta) in EVL cells expressing p120EE-GFP (green), aPKC-delN, wee1-RFP (nuclear red), cdc25, XIAP and stained with phalloidin (cortical red). The dashed white line indicates the surface of the embryo. Scale bars, 10 $\mu$m. Note that there is no cleaved Cas3 staining in the extruding vSrc cells. **(B)** vSrc interferes with the cell cycle, adherens junctions and nuclear composition as well as polarity, leading to apico-basal extrusion. The top panel illustrates a cell undergoing mitosis; the bottom panel, a cell where vSrc hijacked the cell cycle regulators. In the vSrc cell, Pp2A is inactivated earlier in the cell cycle, but counteracting Cdk1 inhibition results in G2/M arrest instead of mitosis. In the vSrc cell, Cyclin B1 (green) is accumulated to a greater extent, the nuclear envelope becomes partially permeable and Anillin (in red) is recruited to adherens junctions by modified p120-catenin. A contractile junctional ring assembles parallel to the plane of epithelium, constricts and releases the cell from the epithelium. vSrc-mediated modulation of the apico-basal polarity complex (e.g. aPKC) promotes the predominantly apical direction of extrusion. Immediate cell death is avoided due to vSrc promoting cell survival.

**Supplementary methods**

**List of primers used**

**To clone the dKrt18 promoter:**
Krt18-0-S1: 5'-CTCGAGGCGCGCCAGTAACACAGCAGAGCAGAGC-3'
Krt18-150-A1: 5'-GAGCTCCAGTAACACAGCAGAGCAGAGC-3'

**To clone from zebrafish gastrula cDNA:**
- Pp2a (ZDB-GENE-050417-441)
PP2A-F: 5'-GTTCTTTTTGCAGGAATGGACGAAAAGGTTTTTACGAAAGAACTG-3
PP2A-R: 5'-TCTAGAGGCTCGAGATTACAGGAAATAATCTGGAGTGCGAC-3'

- RhoA (ZDB-GENE-040426-2150)
RhoA-F: 5'-GTTCTTTTTGCAGGAATGGCTGCGATTCGTAAGAAG-3'
RhoA-R: 5'-TCTAGAGGCTCGAGACTATAGCAAAGCGCAGGCATT-3'

- CcnB1 (ZDB-GENE-000406-10)
Ccnb-EcoRI-F: 5'-CCATCGATTCGAATTATGATGGCTCTCCGTGTCACA-3'
Ccnb-EcoRI-R: 5'-AGTAGGCCTTGAATTTGAGATCTGCTTAGCCAGGTC-3'

- Cdk1 (ZDB-GENE-010320-1)
CDK1-InF-F: 5'-GTTCTTTTTGCAGGAATGGATGACTATCTGAAGATAG-3'
CDK1-InF-R: 5'-TCTAGAGGCTCGAGATTATATCTTGAGGTTGCTGGC-3'

- XIAP (ZDB-GENE-030825-7)
Xiap-S1: 5'-ATGGCACACTCAACTCATAAT-3'
Xiap-A1: 5'-TTAGGCATTGTAAGTCTTTAT-3'

**To create indicated point mutations and deletions:**
- Y307F in CA-Pp2a
PP2A-Y307F-F: 5'-CGCACTCCAGATTTTTTCCTGTAATCT-3'
PP2A-Y307F-R: 5'-AGATTACAGGAAAAAATCTGGAGTGCG-3'

- Q63L in CA-RhoA
RhoA-CA-F: 5'-ATACAGCTGGCCTAGAGGACTACGAC-3'
RhoA-CA-R: 5'-GTCGTAGTCCTCTAGGCCAGCTGTAT-3'

- T19N in DN-RhoA
RhoA-DN-2-F: 5'-AACTGTTTACTCATCGTTTTCAGTAAAGACC-3'
RhoA-DN-2-R: 5'-GATGAGTAAACAGTTCTTTCCACAGGCTCCATCT-3'

- T14A and Y15F in CA-Cdk1
T14AY15F-InF-F: 5'-GGTGCGTTTGGTGTAGTATATAAGGGCAGGAATAAA-3'
T14AY15F-InF-R: 5'-TACACCAAACGCACCTTCACCAATTTTCTCTATCTT-3'

- $\Delta$(1-740) in DN-Anillin
Anillin-DN-F: 5'-GTTCTTTTTGCAGGAAGCTCCAGTCACATCAACATC-3'

p120-del346-R: 5’-TCCTGCAAAAAGAACAAGTAGCTTGTATTC-3’

- Δ(201-591) in DN-aPKC

aPKC-RD-F: 5’-GAACGATGGTCTCGAGAAGCTGAACCCTCCT-3’
aPKC-RD-R: 5’-TCGAGACCATCGTTCTTGTCATCTACTGGAG-3’
TCTAGAGGCTCGAGA

- Y417F in aPKC-mutF

aPKC-Y417F-F: 5’-CCGAACTTTATCGCCCCCGAAATCC-3’
aPKC-Y417F-R: 5’-GGCGATAAAGTTCGGGGTTCCACA-3’

- Δ(1-201) in aPKC-delN

aPKC-delN-F: 5’-
GTTCTTTTTGCAGGAATGGTAGACCTTCCTTCAGAAGAAACTGATG-3’
p120-del346-R: 5’-TCCTGCAAAAAGAACAAGTAGCTTGTATTC-3’

- Y217,228F in p120-mutFF

p120_Y217_Y228F_F: 5’-GGTTATCCAGGTGGCAGTGACAACTtCGGCAGT-3’
p120_Y217_Y228F_R: 5’-GCCACCTGGATAACCATCTTCAaAGTGTCGGC-3’

- Y217,228E in p120-mutEE

p120_Y217,228E_F: 5’-GGTTATCCAGGTGGCAGTGACAACGAAGGCAGTCTGTCC-3’
p120_Y217,228E_R: 5’-GCCACCTGGATAACCATCTTCTTCGTGTCGGCCATACCC-3’

- Δ(375-1127) in DN-ParD3

Par3DN-R: 5’-TCCTGAGCTCCCTCCATTATGGGATATCTGCTCATACTGGGAC-3’
EGFP-Par3DN-F: 5’-GGAGGGAGCTCAGGAATGGTGAGCAAGGGCGAGG-3’

**To create nucGFP:**

nucGFP was created by fusing 2xNLS Sv40 with NLS from Wee1 (RNNRKRSHWN), hmAzami-Green and EGFP.
NLS-AG-F: 5’-
CCAAAGAAGAAGCGTAAGGTAAATCGATCGATGGTGAGCGTGATCAAGCCC-3’
NLS-AG-R: 5’-TCCTGATCCACCACTTCCCTTGGCCTGGCTGGGCAG-3’
NLS-fix-F: 5’-AGAAGCGTAAGGTAAATCGATCGGGGGTGAGCGTGA-3’
NLS-fix-R: 5’-TTACCTTACGCttCTTCTTTGGCATGAATCGATGGG-3’
Alt-NLS-F: 5’-
CCGAAGAAAAAAAGGAAAGTGGACCCAAAGAAGAAGCGTAAGGTAAATC-3’
Alt-NLS-R: 5’-CCTTTTTTTCTTCGGACTTCCcatGAATCGATGGGATCCTGCAAA-3’
NLS-wee1-F: 5’-CAGGAAACGATCACACTGGAATAGTGGTGGAGGGAGCTCAGGA-3’
NLS-wee1-R: 5’- TGTGATCGTTTCCTGTTGTTTCTTCCGCCACCTCCTGATCCA-3’

**To create CDK1pep:**

mKO2-FLAG-F: 5’-TACAAGGATGACGACGATAAGGTGAGTGTGATTAAACCAGAG-3’
CS2-FLAG-R: 5’-GTCGTCATCCTTGTAATCCATTCCTGCAAAAAGAACAAGTAG-3’
CB1-CDK1pep-F: 5’-
GGAGAAGGTACCTATGGAGTTGTGTATAAGAGTGGTGGAGGGAGCTCA-3’

mKO2-CKD1pep-R: 5’-ATAGGTACCTTCTCCAATTTTCTCTATTTTTCCGCCACCTCCTGATCC-3’
CDK1pep-HA-F: 5’-TGACTATGCAGGCCTGTAATCTCGAGCCTCTAGAAC-3’
CDK1pep-HA-R: 5’-AACATCGTATGGATAGATCCCTCCACCACTCTT-3’
2xHA-PCR-F: 5’-GATCTATCCATACGATGTTCCAGAT-3’
2xHA-PCR-R: 5’-ATTACAGGCCTGCATAGTCAGGG-3’

**To create a variety of bidirectional pBR-Tol2-dUAS constructs** we used two unique restriction sites (PmeI and EcoRV) on either side of the dUAS element. We cloned multiple CDS sequences into either of them from the pCS2 vector using primers:
dUAS-PmeI-IF-S1: 5’-TAATGAGCTCGGTTTGGAGCAAGCTTGATTTAGGTG-3’
dUAS-PmeI-IF-A1: 5’-GGATCGAATTCGTTTCCGCGAATTAAAAAACCTCCC-3’
dUAS-EcoRV-IF-S2: 5’-GATCCTGGTACCGATTTGATTTAGGTGACACTATAG-3’
dUAS-EcoRV-IF-A2: 5’-CCGCAAGTCGACGATCCGCGAATTAAAAAACCTCCC-3’

**To create pBR-Tol2-UAS constructs** we used a unique restriction site (EcoRV). We cloned CDS sequences from the pCS2 vector using primers dUAS-EcoRV-IF-S2 and dUAS-EcoRV-IF-A2.

**To create bidirectional pBR-Tol2-dKrt18 constructs** we used two unique restriction sites (PmeI and AscI) on either side of the dUAS element. We cloned multiple CDS sequences into either of them from the pCS2 vector using primers:
Krt18-PmeI-IF-S1: 5’-AGCTCGGATCCGTTTGGAGCAAGCTTGATTTAGGTG-3’
Krt18-PmeI-IF-A1: 5’-GCCGCGTCGACGTTTCCGCGAATTAAAAAACCTCCC-3’
Krt18-AscI-IF-S2: 5’-TGTGTTACTGGCGCGGGAGCAAGCTTGATTTAGGTG-3’
Krt18-AscI-IF-A2: 5’-CGCGCCTCGAGGGCCCCGCGAATTAAAAAACCTCCC-3’

**To create pBR-Tol2-Krt18 constructs** we used a unique restriction site (PmeI). We cloned CDS sequences along with Poly A signal from the pCS2 vector with primers Krt18-PmeI-IF-S1 and Krt18-AscI-IF-S2 using the In-Fusion cloning system (Takara Bio).

## Supplementary references


1 Buckley, C. E. *et al.* Mirror-symmetric microtubule assembly and cell interactions drive lumen formation in the zebrafish neural rod. *EMBO J* **32**, 30-44, doi:10.1038/emboj.2012.305 (2013).
