# Supplementary material for: Src-transformed cells hijack mitosis to extrude from the epithelium
Source: Nat Commun. 2018 Nov 8;9:4695. doi: 10.1038/s41467-018-07163-4 (PMC6224566; doi:10.1038/s41467-018-07163-4)
Supplement: Supplementary file 3 — Description of Additional Supplementary Files [file 41467_2018_7163_MOESM3_ESM.pdf]

Supplementary Movies

Supplementary Movie 1. Time-lapse imaging of vSrc cell extrusion from the EVL of the zebrafish embryo. Transgenic embryos obtained from a line expressing an RFP-actin marker (red) specifically in the EVL (Krt18:Lifeact-Ruby) line crossed with the Krt18:KalTA4-ERT2 line were injected with the UAS:EGFP-vSrc construct (green). Movies were taken over 4 hours.

Supplementary Movie 2. Time-lapse imaging of vSrc cell extrusion (xz view). The surface function was used to segment GFP-positive Src cells over time using the Imaris software. In this cross section of the embryo, a cell is undergoing an apico-basal split.

Supplementary Movie 3. Time-lapse imaging of Anillin-GFP in mitosis. Embryos were injected with the dUAS:myr-Cherry;Anillin-GFP construct. Movies were taken over 4 hours.

Supplementary Movie 4. Time-lapse imaging of Anillin-GFP during Src cell extrusion. Embryos were injected with the dUAS:myr-Cherry-vSrc;Anillin-GFP construct. Movies were taken over 4 hours.

Supplementary Movie 5 and 6. Time-lapse imaging of H2B-GFP in mitosis and extrusion. Embryos were injected with the dUAS:myr-Cherry-vSrc;H2B-GFP construct. Movies were taken over 4 hours. Movies represent merged channels (5) and green channel only (6).

Supplementary Movie 7 and 8. Time-lapse imaging of CyclinB1-GFP in cell extrusion. Transgenic embryos obtained from a line expressing a cell cycle progression marker specifically in the EVL (Krt18:CcnB1-GFP) crossed with the Krt18:KalTA4-ERT2 line were injected with the construct UAS:myr-Cherry (Movie 7) or UAS:myr-Cherry-vSrc (Movie 8). Movies were taken over 8 hours.

Supplementary Movie 9. Time-lapse imaging of the effect of phospho-mimetic p120-mutEE on the localization of Anillin-GFP in cells arrested at the G2/M transition. Embryos were injected with a combination of the following constructs: dUAS:Cherry-Wee1;CA-Cdc25 and dUAS:p120-mutEE;AnillinGFP. Movies were taken over 4 hours.

Supplementary Movie 10. Time-lapse imaging of vSrc-like extrusion induced by coexpression of p120-mutEE, myr-aPKC and the apoptotic inhibitor XIAP in G2/M-arrested cells. Embryos were injected with a combination of the following constructs: dUAS:Cherry-Wee1;CA-Cdc25, dUAS:p120-mutFF;aPKC-delN and Krt18:XIAP. Movies were taken over 6 hours.

Supplementary Movie 11. Time-lapse imaging of vSrc-like-cell extrusion in (C) segmented using the Imaris software. The surface function was used to segment GFP positive cells over time. In this cross section of the embryo (xz view).
